# Transcriptomics and functional characterization identify *CYP73A16* as a key regulator of flavonoid biosynthesis in mulberry leaves

**DOI:** 10.1186/s12870-026-08804-3

**Published:** 2026-05-02

**Authors:** Rurou Long, Xueying Jin, Michael Ackah, Frank Kwarteng Amoako, Frank Kwekucher Ackah, Frank Kumi, Jianbin Li, Hao Wei, John Nelson Buah, Mengdi Zhao, Weiguo Zhao

**Affiliations:** 1 Jiangsu Key Laboratory of Sericulture Biology and Biotechnology, School of Biotechnology, Jiangsu University of Science and Technology, Zhenjiang, 212100 People’s Republic of China; 2 Key Laboratory of Silkworm and Mulberry Genetic Improvement, Ministry of Agriculture and Rural Affairs, The Sericultural Research Institute, Chinese Academy of Agricultural Sciences, Zhenjiang, 212100 People’s Republic of China; 3 Institute of Plant Nutrition and Soil Science, Kiel University, Hermann- Rodewald-Straße 2, Kiel, 24118 Germany; 4 Department of Molecular Biology and Biotechnology, School of Biological Sciences, College of Agriculture and Natural Sciences, University of Cape Coast, PMB, Cape Coast, Ghana; 5 Department of Crop Science, School of Agriculture, College of Agriculture and Natural Sciences, University of Cape Coast, PMB, Cape Coast, Ghana; 6 Department of Materials Science and Engineering, Suzhou University of Science and Technology, Suzhou, 215011 P. R. China

**Keywords:** Mulberry plant, Transcriptomics, Flavonoid biosynthesis, *CYP73A16*, Gene silencing

## Abstract

**Background:**

Mulberry cultivars are well known for their flavonoid content. However, flavonoid content and the key genes involved in flavonoid biosynthesis in mulberry cultivars Zhong Shen 1 Hao (ZS) and Lv Shenzi (LSZ) are not well documented. This study aimed to analyze the flavonoid content and the key genes involved in flavonoid biosynthesis in ZS and LSZ cultivars. In this study, biochemical, transcriptomic, and functional analyses via virus-induced gene silencing (VIGS) were performed to analyze flavonoid content and key genes involved in flavonoid biosynthesis in ZS and LSZ.

**Results:**

The content of flavonoids in LSZ is higher than in ZS, increasing by 38.1% compared to ZS. Transcriptome analysis identified 1938 differentially expressed genes (DEGs), with 1124 downregulated and 814 upregulated, and 14 DEGs were identified as key genes significantly enriched in flavonoid biosynthesis. Transient silencing of *MaCYP73A16* notably decreased not only the expression of downstream genes, including *MaCYP73A16*,* MaLAR*,* MaCHS*,* MaCYP93B1*, and *MaCYP98A2*, but also reduced flavonoid content, with peak silencing efficiency observed on the ninth day, underscoring the impact of silencing *MaCYP73A16* on other genes involved in the flavonoid biosynthesis pathway.

**Conclusion:**

This study provides a fundamental overview of flavonoid biosynthesis in LSZ and ZS and the key genes involved, which will help facilitate a comprehensive analysis of this pathway and provide significant insight into the large-scale biosynthesis of medicinal flavonoids, thereby improving flavonoid content in these cultivars.

**Supplementary Information:**

The online version contains supplementary material available at 10.1186/s12870-026-08804-3.

## Introduction

The genus *Morus* encompasses mulberry plants, with *Morus alba* L. (white mulberry), *Morus nigra* L. (black mulberry), and *Morus rubra* L. (red mulberry) representing the primary species. This genus is characterized by taxonomic complexity due to prevalent hybridization, leading to ongoing debate over true species numbers within existing germplasms. All species have significant industrial value, with *M*. *alba* being particularly notable. This perennial woody plant of the *Moraceae* family has a long history of use in traditional Chinese medicine and is a key feedstock for silkworms [1, 2]. The medicinal and pharmacological properties of mulberry leaves are attributed to their rich composition of active compounds. These include vitamins, amino acids, polysaccharides, flavonoids, alkaloids, and numerous essential microelements [3–5]. These compounds demonstrate synergistic health benefits, such as improving liver and eye health, lowering blood pressure, regulating blood lipid and sugar levels, and countering hypertension and viral infections [6–8]. The active components, especially the high flavonoid content, are responsible for anti-hypertensive, hypolipidemic, anti-oxidative, antidiabetic, anti-obesity, and anti-inflammatory effects [9, 10]. Notably, mono- and di-O-glycosylated flavonols are the most abundant flavonoids identified in the leaves, and these key metabolites include isoquercitrin, astragalin, kaempferol, quercetin 3-(6-acetylglucoside), and rutin [11, 12].

Flavonoids, a class of phenolic compounds, are structurally categorized into groups such as flavonols, flavones, anthocyanins, flavanols, flavanones, and isoflavones [9, 13]. Their strong biological activity significantly reduces the risk of chronic diseases, including coronary heart disease, cancer, and type II diabetes [10, 14, 15]. This antioxidant function arises from the phenolic hydroxyl structure within polyphenols. This structure oxidizes into a quinone, which consumes oxygen and captures reactive oxygen species (ROS) and other lipid peroxidation by-products [16]. In green tea, for instance, the meta 5,7-dihydroxyl structure of polyphenols and the di- and tri-hydroxyl structures of the B- and D-rings enable various antioxidant activities [17, 18]. Furthermore, the pentahydroxy structure of quercetin confers anti-inflammatory and anti-cancer properties against *Staphylococcus aureus* by targeting ClpP to protect mice from MRSA induced lethal pneumonia [17, 18]. In mulberry, metabolic profiling of 91 resources identified 44 flavonoid compounds. This analysis demonstrated that O-rhamnosylated flavonols and malonylated flavonol glycosides, including rutin and quercetin 3-O-(6″ O malonylglucoside), were absent in *M. notabilis* and several *M. alba* resources [19]. Supporting this, a transcriptome and phylogenetic study highlighted the flavonol 3-O-glucoside-O-rhamnosyltransferase KT324624 as a key enzyme for rutin synthesis in the flavonoid pathway [19]. Flavonoid biosynthesis is influenced by environmental factors like low temperature, drought, and UV radiation, as well as harvest time, processing methods, and hormone induction. Crucially, it is also governed by genetic and cultivar differences. These determinants induce structural enzyme coding genes such as phenylalanine ammonia-lyase (*PAL*), chalcone synthase (*CHS*), chalcone isomerase (*CHI*), flavanone 3 hydroxylase (*F3H*), flavonoid 3’ hydroxylase (*F3’H*), flavonoid 3’5’ hydroxylase (*F3’5’H*), and dihydroflavonol 4-reductase (*DFR*) [20, 21]. They also activate regulatory transcription factors like myeloblastosis domain protein (*MYB*), basic helix loop helix (*bHLH*), and WD40 repeat protein (*WD40*), which act as master switches controlling the expression of these structural genes [20, 21]. For example, overexpression in tobacco leaves led to a significant reduction in anthocyanin content, while total flavonoids remained largely unchanged except in one line, *OE-F3H#2*, which showed a marked decrease [22]. Additionally, cytochrome P450 monooxygenase (CYP) genes are reported as crucial for flavonoid biosynthesis in fruit, playing a vital role in modifying the flavonoid backbone [21, 23].

Cytochrome P450 monooxygenases (CYP) are a multigene superfamily of heme-thiolate proteins with ancient origins. The name P450 derives from the characteristic 450 nm absorption peak of their carbon monoxide binding form [21, 23–25]. As one of the largest enzyme superfamilies, they are ubiquitous across eukaryotes and prokaryotes and constitute approximately 1% of all genes in a plant genome, underscoring their fundamental role in plant development [26–28]. These membrane-bound enzymes are localized to the endoplasmic reticulum via an N-terminal hydrophobic signal sequence and utilize oxygen and NADPH as primary substrates [29] to catalyze a diverse array of reactions, including decarboxylation, epoxidation, sulfoxidation, dehalogenation, dealkylation, hydroxylation, C-C bond cleavage, ring extension, deamination, and reduction [24, 30]. In mulberry, families such as *CYP80*, *CYP92*, *CYP728*, *CYP733*, *CYP736*, and *CYP749* are known to be critical for the biosynthesis of secondary metabolites [27]. P450 genes are integral to flavonoid biosynthesis, with the CYP75 family encoding key enzymes like flavonoid 3’ hydroxylase (F3′H) and flavonoid 3’5’ hydroxylase (F3’5’H) [23, 31]. This functional diversity is illustrated across species. In the sacred lotus, *CYP719* and *CYP80* family genes direct the synthesis of benzylisoquinoline and aporphine alkaloids [28]. In *Medicago sativa* L. the *CYP93C* subfamily participates in isoflavone biosynthesis [32], while in *Hordeum vulgare* L., the *CYP75* family facilitates anthocyanin synthesis, yielding diverse metabolites in different plant tissues [33]. Research in *M. alba* has identified *MaCYP71BG22* as a catalytically specific enzyme. It was cloned and shown to catalyze the stereoselective hydroxylation of (R)-2-methylpiperidine at the C4-position to produce (2R, 4R)-2-methylpiperidin-4-ol [25]. Functional studies in mulberry hairy roots revealed that overexpression of *MaCYP71BG22* significantly elevated the content of 1-deoxynojirimycin (DNJ), a primary active component in mulberry leaves, whereas virus-induced gene silencing (VIGS) reduced its biosynthesis [25], underscoring the diverse functionality of this compound in plants and highlighting the need for further research to unlock its potential.

While numerous studies document the crucial role of CYP450 family genes in mulberry growth and flavonoid accumulation, the functional characterization of the transcriptome-identified gene *CYP73A16* remains unexplored. Significant variation in flavonoid biosynthesis and content exists among different plant cultivars, a determinant that can guide the identification of resources rich in specific beneficial compounds. We hypothesize that cultivar and genotype variation in mulberry induces differential flavonoid biosynthesis, orchestrated in part by the *CYP73A16* gene, which catalyzes the production of varying flavonoid classes and accumulation levels. This study investigated flavonoid accumulation in the leaves of two mulberry cultivars, Zhong Shen 1 Hao (ZS) and Lv Shenzi (LSZ). Through parallel transcriptomic analysis, we identified key flavonoid biosynthesis genes, including CYP family genes, and functionally characterized *MaCYP73A16* using gene silencing. This approach elucidated its involvement in the molecular mechanisms and pathways of flavonoid biosynthesis. By identifying key flavonoid biosynthesis genes via transcriptomics and the functional validation of *MaCYP73A16*, this work provides a new perspective on the biological role of *MaCYP73A16* in mulberry flavonoid biosynthesis, which may contribute to its efficient production and commercial application.

## Materials and methods

### Mulberry materials and growth conditions

Mulberry Zhong Shen 1 Hao (ZS) and Lv Shenzi (LSZ) were sourced from the National Mulberry GenBank at Jiangsu University of Science and Technology, Zhenjiang, China. The ZS and LSZ are planted at the Institute of Sericulture, Chinese Academy of Agricultural Sciences, Jiangsu University of Science and Technology, Zhenjiang City, Jiangsu Province, China (32 ° 11 ′ 45.80 ″ N, 119 ° 23 ′ 45.80 ′ E). Zhong Shen 1 Hao and Lv Shenzi used in this study were 10 years old, with an average height of 2 m and a stem diameter of 5 cm. They were maintained under controlled environmental conditions: a temperature of 9–25 ℃, a relative humidity of 40–60%, and a 12-h photoperiod at a light intensity of 15,000 lx. Irrigation was supplied naturally via rainfall. The leaves of these plants at the vegetative stage were plucked during the daytime (3 p.m.) and used for flavonoid content and transcriptome analyses. Due to limited study materials for ZS and LSZ (including no mutants generated yet and no seeds available to produce seedlings for a VIGS experiment), Yu-711 seeds were used for the functional validation. The mulberry seedlings (Yu-711) maintained in Prof. Zhao Weiguo’s laboratory at Jiangsu University of Science and Technology were used to construct the *MaCYP73A16* cloning vector and the VIGS vector, and for downstream post-silencing measurements.

### Determination of total flavonoid content

Total flavonoid content was analyzed in leaves from ZS and LSZ plants using a plant flavonoids test kit (M0118A, Suzhou Michy Biomedical Technology Co., Ltd., China). Following the manufacturer’s instructions, leaf samples were freeze-dried to constant weight, ground, and sieved (40-mesh). Approximately 0.05 g of powder was used for the determination. Analyses were performed with three biological and three technical replicates per sample group.

### RNA extraction, library construction, and sequencing

Total RNA was extracted from leaves of ZS and LSZ plants (three biological replicates per genotype) using TRIzol reagent (Invitrogen, USA). RNA integrity and quality were assessed by agarose gel electrophoresis and an Agilent 2100 Bioanalyzer (Agilent Technologies, USA). cDNA was prepared according to our previous work [34] using the NEBNext Ultra RNA Library Prep Kit for Illumina (NEB, USA). The final cDNA libraries were sequenced on an Illumina Novaseq 6000 platform at Gene Denovo Biotechnology Co. (Guangzhou, China).

### Bioinformatics and data analysis of the RNA-seq results

Raw sequencing reads were processed using fastp (v0.18.0) [34] to obtain clean reads after filtering adapter sequences, low-quality reads, and unknown nucleotides. Residual ribosomal RNA (rRNA) reads were filtered by alignment to an rRNA database using Bowtie2 (v2.2.8) [35]. The resulting high-quality, rRNA-depleted reads were mapped to the *M. notabilis* reference genome (https://morus.biodb.org/index) using HISAT2 (v2.4) [36]. Gene expression was quantified via a reference-based assembly approach using StringTie (v1.3.1) [37, 38], and transcript abundances were calculated as TPM (Transcripts per Million mapped reads) using RSEM (v1.3.3) [39]. Sample reproducibility was assessed by correlation and Principal Component Analysis (PCA) in the R package gmodels (http://www.rproject.org/) (v4.2). DESeq2 (v1.42.1) [40] was used to identify differentially expressed genes (DEGs) with |log2(fold change) | ≥ 1 and FDR ≤ 0.05. DEGs were visualized using volcano plots and heatmaps in R (v4.2). Functional enrichment for GO terms and KEGG pathways was considered significant at FDR ≤ 0.05.

### Validation of the RNA-seq results by qRT-PCR

Fourteen DEGs were chosen for qRT-PCR validation based on their expression and relevance to key GO terms and KEGG pathways. Total RNA from the same ZS and LSZ leaves was reverse transcribed into cDNA, and qRT-PCR was conducted as previously reported [41] with gene-specific primers (Table S1). Relative expression was calculated using the 2^−ΔΔCt^ method [42].

### Molecular cloning and VIGS vector construction for *MaCYP73A16*

*M. alba* (car. Yu-711) seeds were used for the cloning and the post-*MaCYP73A16* silencing total flavonoid determination due to the limited materials availability of ZS and LSZ, and to its closeness with the cultivars used, as revealed by phylogeny analysis. The *MaCYP73A16* gene (XP_010103664.1, trans-cinnamate 4-monooxygenase) was identified in our transcriptome analysis of ZS and LSZ leaves and selected for validation based on its expression level and involvement in the flavonoid biosynthesis pathway. Using the SGN VIGS website (https://vigs.solgenomics.net/), a silencing fragment with high specificity for the target gene, with a silencing length set at 200–300 bp, was predicted from the coding sequence (CDS) of the *CYP73A16* gene. The gene was PCR-amplified using gene-specific primers: MaCYP73A16-F:5’-ATGGACCTCCTCTTCCTAGAGAA-3’ as the forward primer and MaCYP73A16-R: 5’-TCAGCACACTCTGGGCTTG-3’ as the reverse primer, as previously described [43]. The amplified product was cloned into the pMD™19-T vector and transformed into DH5α cells (Nanjing Vazyme Biotech Co., Ltd.), which were plated on Luria-broth: LB-ampicillin agar (1 g of yeast extract, 2 g of tryptone, 2 g of sodium chloride, and 3 g of agar powder, added to 200 mL distilled water, 20 µl of ampicillin per plate). After 14–16 h of incubation at 37 °C, positive clones were identified by PCR and sequenced (Zhejiang Shangya Biotechnology). The construction of VIGS vector followed the same procedure as in our previous study [43]. A total of 30 mulberry seedlings, raised as previously described in our study [43] and exhibiting similar growth characteristics, were selected and divided into control (15 seedlings) and experimental groups (15 seedlings), one seedling per pot, in a completely randomized design. The pNC-TRV2-GFP vector was digested with SfiI (Thermo Fisher) overnight.

The *MaCYP73A16* silencing fragment was amplified using primers MaCYP73A16-TRV2-F:5’-agtggtctctgtccagtcctGTGGTGGCTGGCCAACAA-3’ as the forward primer and MaCYP73A16-TRV2-R:5’-ggtctcagcagaccacaagtGCAACAATAGTGGAGTGCTTCAA-3’ as the reverse primer. Also, the pTRV2-GFP- F: 5’-atggtgagcaagggcgag-3’ as the forward primer and pTRV2-GFP-R: 5’-ttacttgtacagctcgtccatgc-3’ as the reverse primer were used as the control vector primers. The inserts were cloned into the linearized vector using seamless cloning. The resulting recombinant plasmid was transformed into DH5α cells, cultured, and verified by PCR and sequencing.

### Plant transformation and gene silencing analysis

The plasmids pTRV2-GFP and pTRV2-GFP-MaCYP73A16 were transformed into *Agrobacterium* GV3101. Four-week-old seedlings were infected by ultrasonically mediated wounding using bacterial suspensions, with three biological replicates. After 36–48 h in darkness, plants were transferred to the growth chamber under the same conditions as described above. GFP fluorescence confirmed transformation. Leaves were sampled at 3, 6, 9, and 12 days post-infection for qPCR analysis. The MaCYP73A16 expression was quantified using gene-specific primers, including MaCYP73A16-RT-F: 5’-TGGCTATCCCTCTGCTGGTG-3’ as the forward primer and MaCYP73A16-RT-R: 5’-TCCCATTGGCCTCAACCTTA-3’ as the reverse primer. The mulberry actin2 gene (HQ163774) was used as an internal reference, with primers F: 5’-GCATGAAGATCAAGGTGGTG-3’ and R: 5’-CATCTGCTGGAAGGTGCTAA-3’. Each sample underwent triplicate biological and technical analysis. Relative expression was calculated by the 2^−ΔΔCt^ method, and statistical significance (*p* < 0.05 or *p* < 0.01) was assessed using Student’s t-test in GraphPad Prism (v9.5.0).

### Subcellular localization of CYP73A16 in the mulberry plant

To investigate the localization of the *CYP73A16* gene experimentally, the plant-mPLoc website (http://www.csbio.sjtu.edu.cn/cgi-bin/PlantmPLoc.cgi) was used to predict its localization, which was predicted to be in the endoplasmic reticulum (ER). To confirm the prediction, tobacco (*Nicotiana benthamiana*) seeds were germinated in nutrient-rich soil and grown under controlled conditions: 24 °C, 60% relative humidity, and a 16‑h light/8-h dark photoperiod in a growth chamber. Thirty uniformly growing plants were selected and grouped into control and experimental groups (15 plants each), with one plant per pot in a completely randomized design. Four‑week‑old, evenly developed seedlings were used for the experiment. *Agrobacterium tumefaciens* strain GV3101 harboring the constructs 35 S: GFP, 35 S:*MaCYP73A16*‑GFP, and the marker gene *AtWAK2* was retrieved from − 80 °C storage and activated to an OD₆₀₀ of 3.0. Cells were harvested by centrifugation at 5,000 rpm for 10 min and resuspended in a buffer containing 10 mM MgCl₂, 10 mM MES (pH 5.6), and 100 µM acetosyringone. After resuspension, the cultures were incubated at room temperature for 3 h.

For infiltration, the control suspension was prepared by mixing equal volumes of *Agrobacterium* carrying 35 S: GFP and *AtWAK2* (marker). The experimental suspension was prepared similarly by mixing equal volumes of *Agrobacterium* carrying 35 S:*MaCYP73A16*‑GFP and *AtWAK2*. The mixed suspensions were infiltrated into tobacco leaves using a needleless syringe. Following infiltration, plants were kept in a dark, humid environment. Two days post‑infiltration, leaf sections (approximately 1 cm²) were excised and mounted for imaging. Fluorescence signals were observed using a confocal laser scanning microscope. GFP was excited at 488 nm with emission detected at 493–591 nm; mCherry (AtWAK2 marker) was excited at 561 nm with emission detected at 585–610 nm; and bright‑field imaging was performed with excitation at 633 nm and emission detected at 647–722 nm.

### Flavonoid content and downstream gene analysis after *MaCYP73A16* silencing

Total flavonoid content in the *M. alba* leaves was determined after silencing the *CYP73A16* gene. This followed the plant flavonoids test kit (item number M0118A) obtained from Suzhou Michy Biomedical Technology Co., Ltd., Suzhou, China. Leaf sample preparation is the same as stated earlier in the methods. The *CYP73A16* and downstream gene expression after silencing were measured by qRT-PCR with primers from Table S1. Gene expression and flavonoid content were assessed using three biological and technical replicates. Statistical significance (*p* < 0.05 or *p* < 0.01) was determined by Student’s t-test in GraphPad Prism (v9.5.0).

### Phylogenetic analysis of CYP73A16 protein sequences

A phylogenetic tree was constructed using the Neighbor-Joining method based on the full-length CYP73A16 protein sequence derived from the transcriptome and the sequences of *M. alba*,* Macadamia integrifolia* Maiden & Betche, *Hevea brasiliensis* Müll. Arg, *Juglans regia* L., *Theobroma cacao* L., and *Jatropha curcas* L derived from NCBI. The analysis was performed in MEGA 12 employing the JTT + G amino acid substitution model. Branch support was evaluated with 1000 bootstrap replicates.

## Results

### Determination of flavonoid content in two mulberry cultivars

**Fig. 1:**
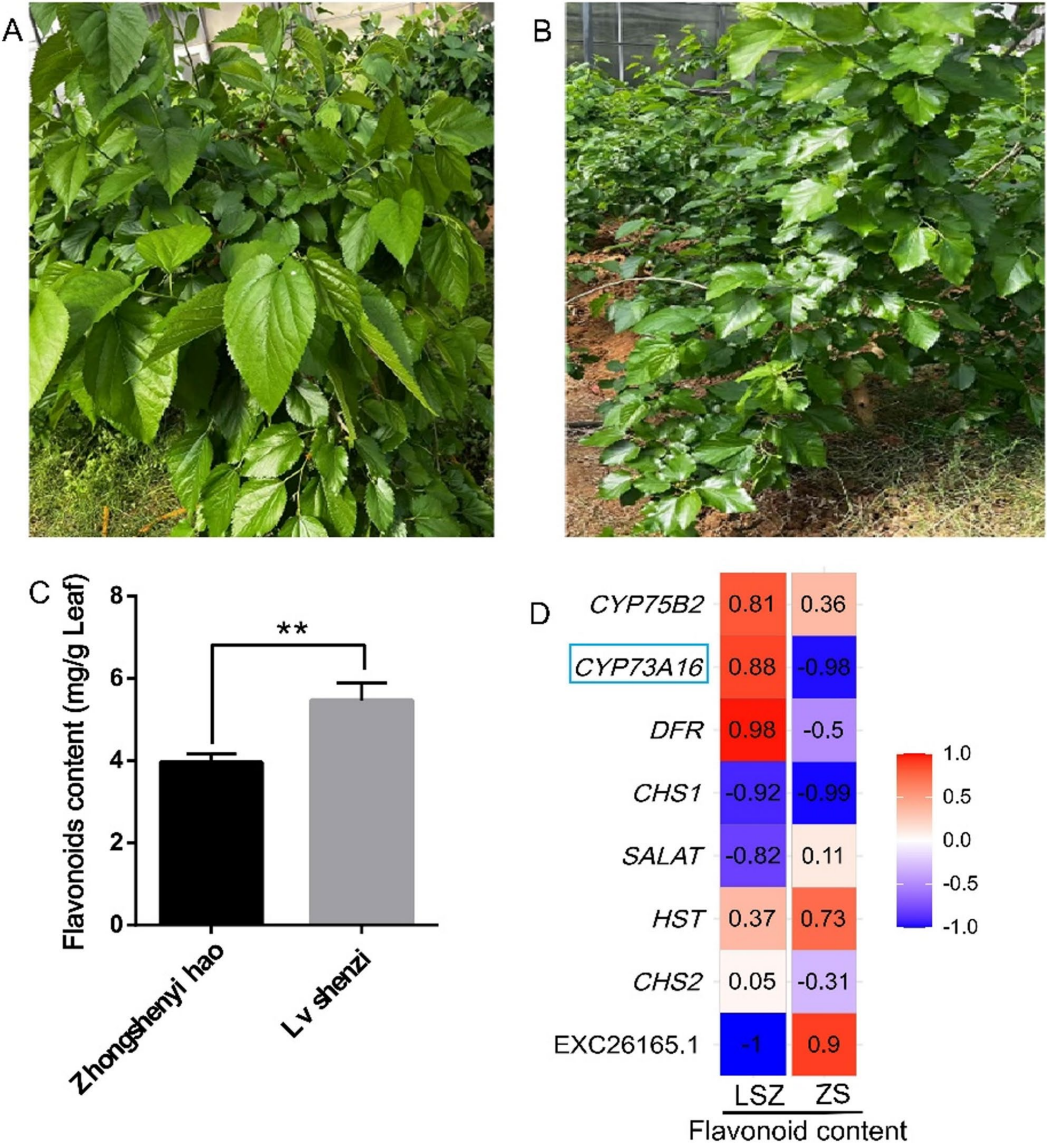
**A** Plant morphology of Zhong Shen 1 Hao (ZS). **B** Plant morphology of Lv Shenzi (LSZ). **C** Total flavonoid contents between Zhong Shen 1 Hao and Lv Shenzi. **D** Correlation plot of some flavonoid biosynthesis genes with flavonoid content in LSZ and ZS. Blue and red indicate negative and positive correlations, respectively. The gene marked with a blue rectangle is the gene used for silencing. Columns are the mean values of three replicates, and error bars represent the standard deviation of the three replicates. Asterisks represent statistical significance at ** *p* < 0.01 using the Student’s t-test

Analysis of total flavonoid content in the leaves of Zhong Shen 1 Hao (ZS) and Lv Shenzi (LSZ) reveals that the flavonoid content in the leaves of LSZ is significantly (*p* ≤ 0.05) higher than in the leaves of ZS (Fig. 1A-C). Compared to ZS, the content of flavonoid increased by 38.1% in LSZ (Fig. 1C).

### Transcriptome profile analysis of Zhong Shen 1 Hao and Lv Shenzi leaves

To understand the molecular mechanism underlying flavonoid biosynthesis in Zhong Shen 1 Hao (ZS) and Lv Shenzi (LSZ) leaves, we performed high-throughput RNA sequencing on the leaves of LSZ and ZS. From the RNA-seq result, 45.67 million raw reads on average were obtained from LSZ, whereas 39.79 million raw reads on average were obtained in ZS (Fig. S1A; Table 1). After filtering the raw reads, 45.56 million clean reads (on average), representing 99.8%, were obtained from LSZ, whereas 39.71 million clean reads, representing 99.8%, were obtained from ZS (Fig. S1B; Table 1). Analysis of the base composition of the clean reads reveals that the GC content of the sample reads (on average) was 46.2%, whereas the Q20 and Q30 scores are 96.8% and 90.9%, respectively (Table S2). By mapping the clean reads to the *M. notabilis* reference genome, 93.5% and 92.6% were uniquely mapped in LSZ and ZS, respectively. Meanwhile, 97% and 95.4% of the clean reads in LSZ and ZS, respectively, were fully mapped to the reference genome (Table S3). Mapping clean reads to the reference genome was predominantly to exons, with 82.3% mapped on average (Fig. S1C). The TPM distribution of the gene and the gene expression density showed an even distribution of reads across sample groups (Fig. S2A, B). Pearson’s correlation heatmap of the samples showed that the correlation coefficient (R^2^) was greater than 0.9, indicating the data’s reliability (Fig. S2C). A clear separation of LSZ and ZS samples was revealed by Principal Component (PC) analysis, with PC1 accounting for 99.7% of the variation (Fig. S2D).

**Table 1:** Sequence statistics and quality control analysis

Sample	Raw Data	Clean data (%)	Low quality (%)	Adapter (%)	poly A (%)	*N* (%)
LSZ-1	44,337,588	44,238,444 (99.78%)	66 (0.00%)	12,808 (0.03%)	0 (0.00%)	86,270 (0.19%)
LSZ-2	47,685,554	47,579,484 (99.78%)	78 (0.00%)	13,932 (0.03%)	0 (0.00%)	92,060 (0.19%)
LSZ-3	44,970,652	44,870,740 (99.78%)	76 (0.00%)	12,614 (0.03%)	0 (0.00%)	87,222 (0.19%)
ZS-1	38,558,548	38,474,966 (99.78%)	60 (0.00%)	10,762 (0.03%)	0 (0.00%)	72,760 (0.19%)
ZS-2	39,139,348	39,061,690 (99.80%)	34 (0.00%)	11,648 (0.03%)	0 (0.00%)	65,976 (0.17%)
ZS-3	41,681,002	41,588,594 (99.78%)	50 (0.00%)	12,102 (0.03%)	0 (0.00%)	80,256 (0.19%)

Raw Data: the total number of reads (sequences) generated directly by the sequencing instrument before any filtering or processing. Clean data (%): the percentage of raw reads remaining after removing low-quality reads, adapters, poly-A sequences, and other contaminants. Low quality (%): the percentage of reads discarded due to poor base quality scores. Adapter (%): the percentage of reads containing adapter sequences (artificial DNA sequences added during library preparation). poly A (%): the percentage of reads consisting primarily of poly-A tails (strings of adenine nucleotides). N (%): the percentage of reads containing ambiguous bases

*LSZ*; Lv Shenzi, *ZS*; Zhong Shen 1 Hao

### Analysis of differentially expressed genes (DEGs) and functional annotations in Zhong Shen 1 Hao and Lv Shenzi leaves

**Fig. 2:**
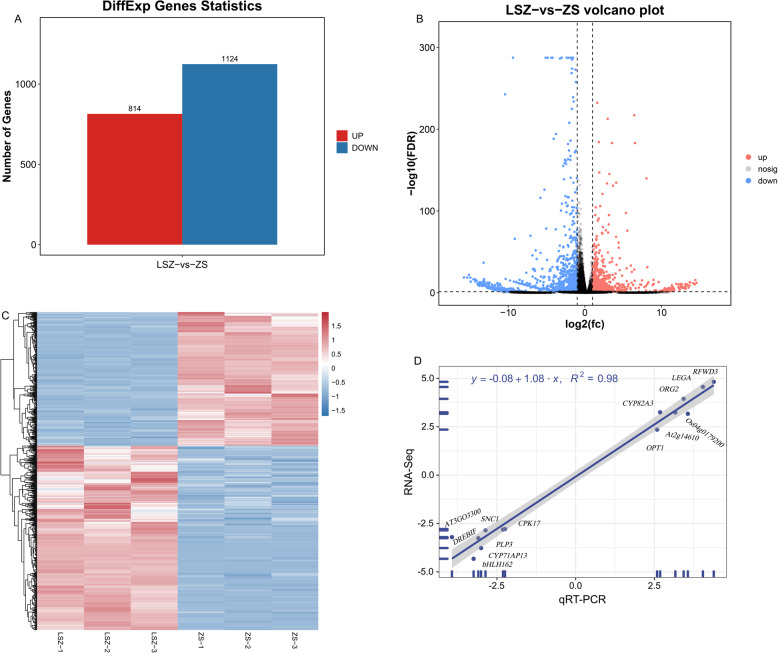
Differentially expressed genes (DEGs) statistics. **A** DEGs statistics and expression pattern. **B** Volcano plot showing the pattern of the DEGs distribution in Zhongshen 1 Hao (ZS) and Lv Shenzi (LSZ). **C** Heatmap showing the DEGs patterns in ZS and LSZ. Red and blue colors in the heatmap and volcano plot indicate up- and downregulation of genes, respectively. **D** Regression analysis showing the validation of DEGs by RT-qPCR. *CYP82A3* (Cytochrome P450 CYP82D47), *OPT1* (Oligopeptide transporter 1), *LEGA* (legumin A), *At2g14610* (hypothetical protein GH714_008727), *ORG2* (transcription factor ORG2), *Os04g0179200* (tropinone reductase-like 2), *RFWD3* (E3 ubiquitin-protein ligase), *At3g03300* (endoribonuclease dicer-2-like), *bHLH162* (transcription factor bHLH162), *DREB1F* (dehydration-responsive element-binding 1E), *CPK17* (calcium-dependent protein kinase 26), *CYP71AP13* (cytochrome P450 71A1), *PLP3* (patatin-like protein 3)

**Fig. 3:**
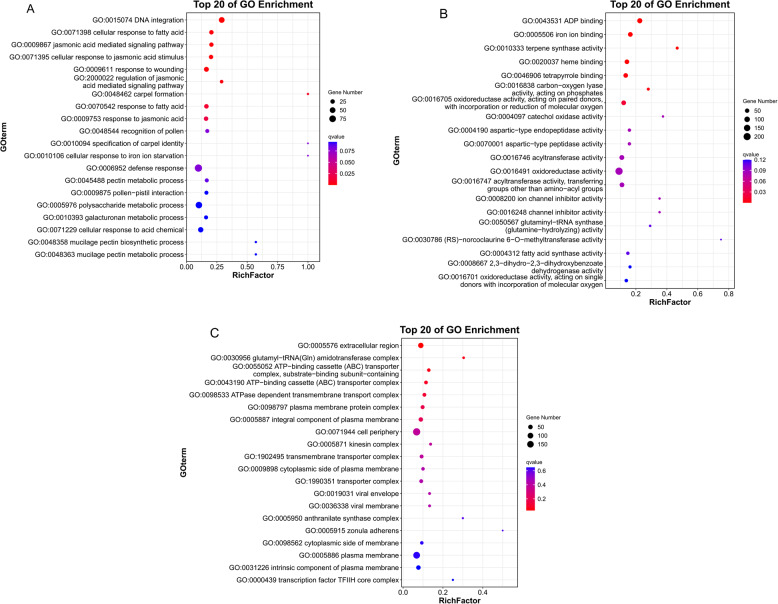
Top 20 gene ontology (GO) functional annotation enrichment analysis of the DEGs. **A**-**C** GO enrichment in biological process, molecular function, and cellular component, respectively. The colors represent the concentration of DEGs. The bubble size indicates the number of DEGs within each GO term

Analysis of LSZ and ZS leaves transcript profiles reveals 25,195 transcript expressions between LSZ and ZS. Of the expression data, 1938 genes were differentially expressed (DEGs), with 1124 downregulated and 814 upregulated in the LSZ-vs-ZS comparison (Fig. 2A). Volcano plot (Fig. 2B) and cluster heatmap analysis (Fig. 2C) reveal contrasting patterns of DEGs between LSZ and ZS. Functional annotation of the DEGs by GO terms reveals that the DEGs were enriched in three GO categories, including biological process (BP; Fig. 3A), Molecular function (MF; Fig. 3B), and cellular component (CC; Fig. 3C). In the BP, DNA integration (51 genes), jasmonic acid mediated signaling pathway (23 genes), cellular response to fatty acid (24 genes), etc. were significantly enriched (Fig. 3A). Again, in the MF, ADP binding (78 genes), iron ion binding (63 genes), terpene synthase activity (14 genes), and many others were significantly enriched (Fig. 3B). Meanwhile, in the CC, extracellular region (84 genes), cell periphery (194 genes), transmembrane transporter complex (30 genes), and others were significantly enriched (Fig. 3C). Analysis of the GO secondary level of the DEGs reveals that they were mostly enriched in signaling, cellular process, metabolic processes, etc. in the BP, whereas cellular anatomical entity, protein-containing complex were enriched with the DEGs in the CC (Fig. S3). Meanwhile, catalytic activity, binding, antioxidant activity, translation regulator activity, etc., were enriched in the MF (Fig. S3).

**Fig. 4:**
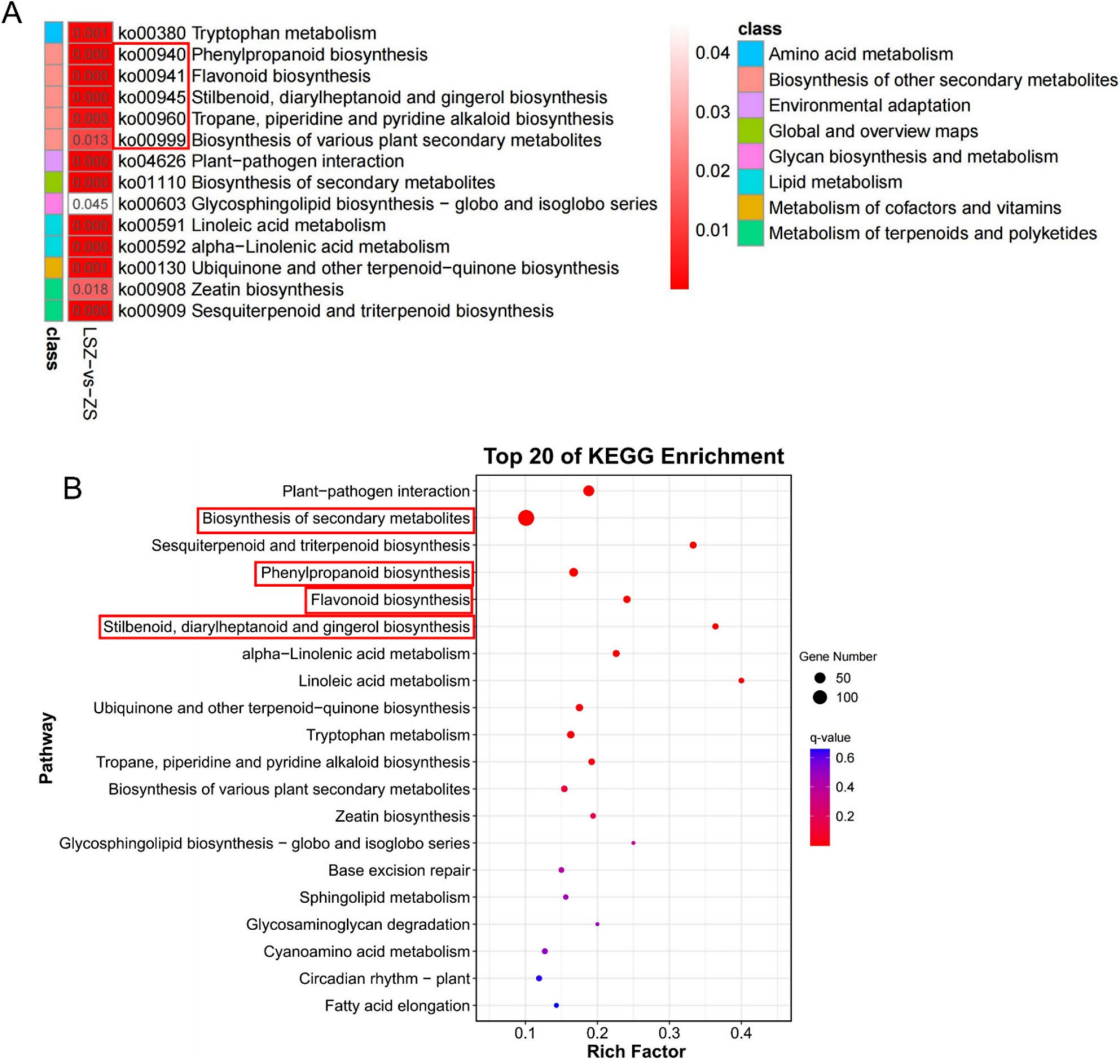
KEGG annotation and classification analysis involving the DEGs. **A** KEGG-pathway classification based on a significant p-value (*p* ≤ 0.05). **B **The top 20 KEGG pathway enrichment involving the DEGs. The colors represent the concentration of DEGs. The bubble size indicates the number of DEGs in each KEGG pathway. Red to blue indicates higher to lower gene expression levels

To know the metabolic pathways of the DEGs, KEGG pathway analysis was performed. Based on the analysis, the DEGs were significantly enriched (*p* ≤ 0.05) in metabolism, spanning 12 classifications. Among these significant pathways are flavonoid biosynthesis (14 genes), phenylpropanoid biosynthesis (25 genes), biosynthesis of secondary metabolites (141 genes), biosynthesis of various plant secondary metabolites (10 genes), sesquiterpenoid and triterpenoid biosynthesis (11 genes), zeatin biosynthesis (6 genes), and many other pathways (Fig. 4A-B).

### Analysis of flavonoid and phenylpropanoid biosynthesis genes

**Fig. 5:**
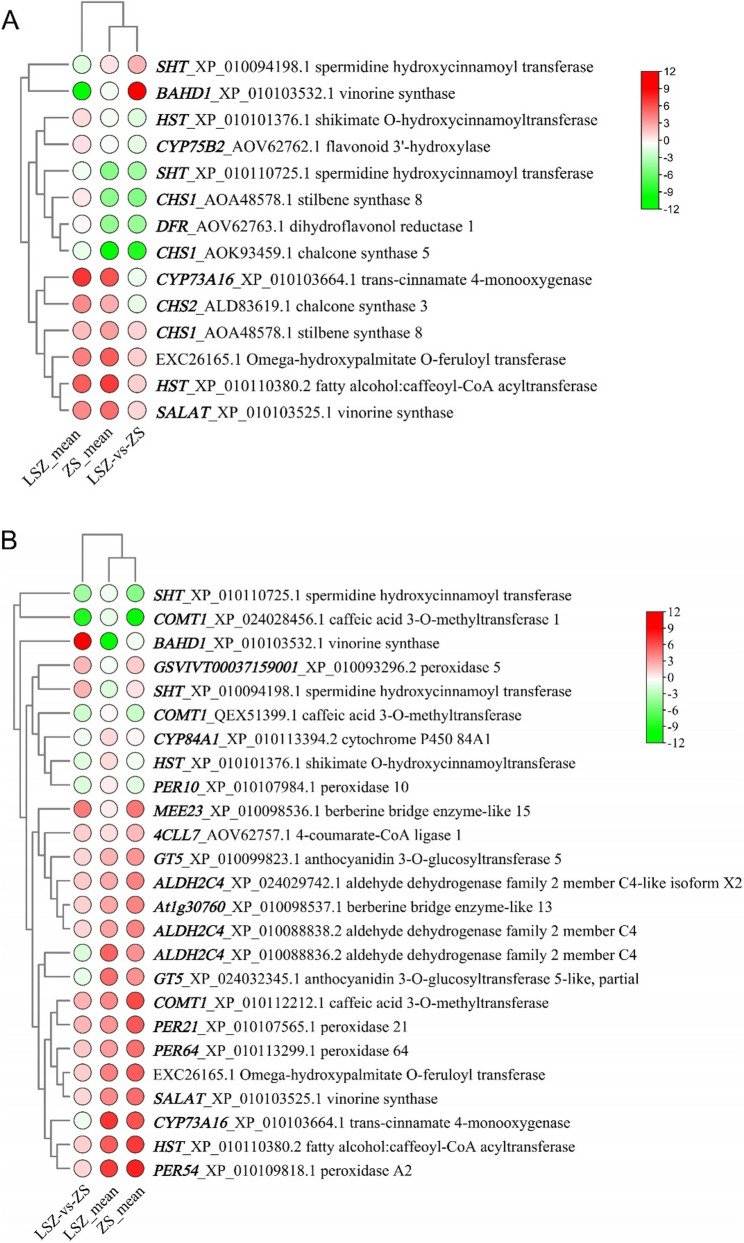
Analysis of DEGs involved in flavonoid and phenylpropanoid pathways. **A** DEGs in the biosynthesis of the flavonoid pathway. **B** Phenylpropanoid pathways. Gene symbols are in bold font and italicized. LSZ_mean indicates the gene expression or abundance in LSZ samples. ZS_mean represents gene expression or abundance in ZS samples. LSZ-vs-ZS represents the differential gene expression (DEGs) between LSZ and ZS. Red to green represents higher to lower gene expression levels

**Fig. 6:**
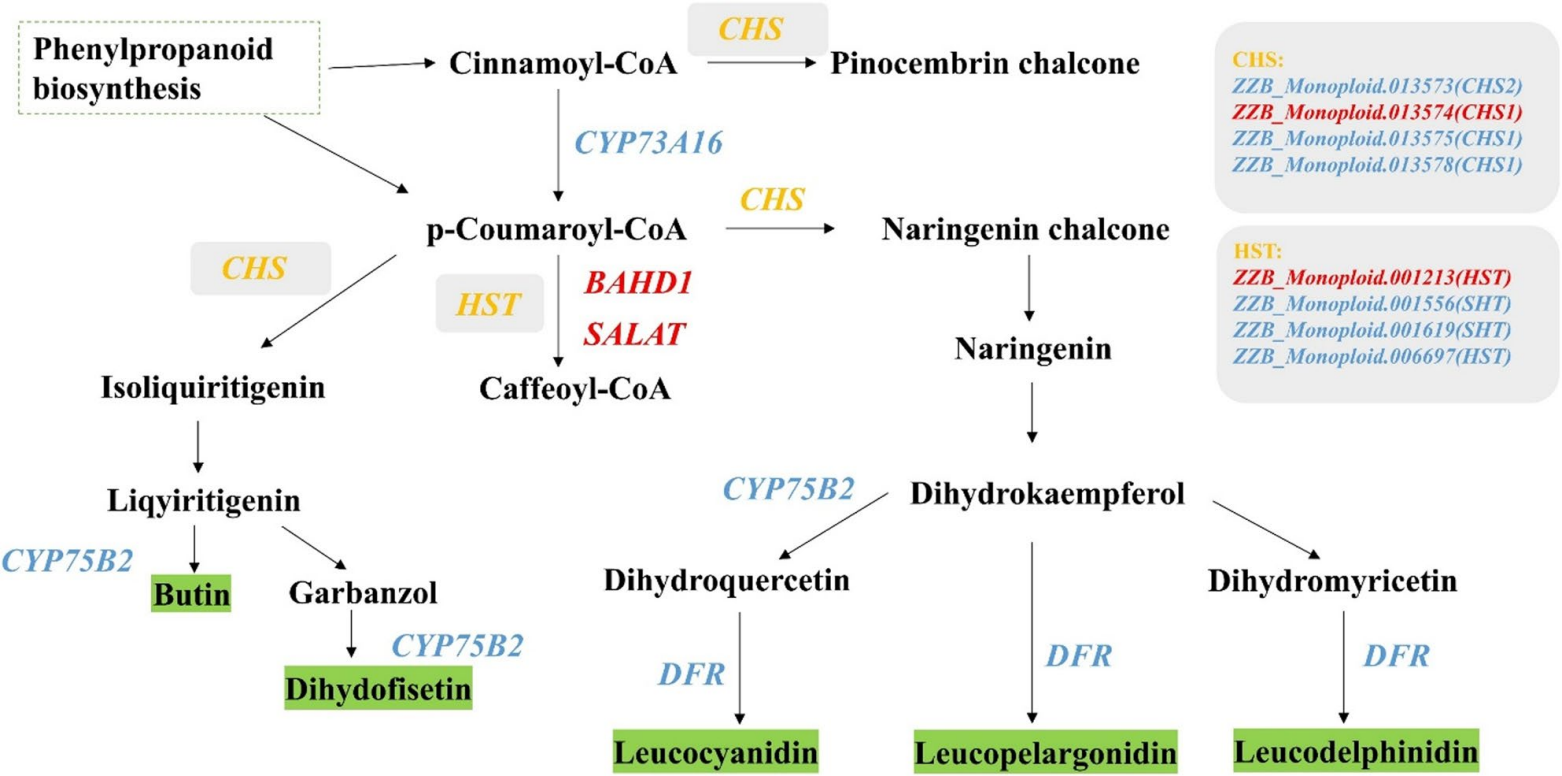
A simplified representation of the flavonoid biosynthetic pathway. Production is derived from a metabolic pathway that begins with the general phenylpropanoid pathway, followed by the flavonoid biosynthetic pathway. This pathway involves several key structural enzymes that lead to the production of butin, dihydofisetin, leucocyanidin, leucopelargonidin and leucodelphinidin, which are highlighted in light green. Gene symbols in blue and red are down- and upregulated, respectively, whereas those in yellow have both up- and downregulation in the pathway. *CHS*; chalcone synthase, *HST*; shikimate O-hydroxycinnamoyltransferase and fatty alcohol: caffeoyl-CoA acyltransferase, *BAHD1*; vinorine synthase, *DFR*; dihydroflavonol reductase 1, *CYP75B2*; flavonoid 3’-hydroxylase, *SALAT*; vinorine synthase, and *CYP73A16*; trans-cinnamate 4-monooxygenase

Analysis of the flavonoid biosynthesis genes reveals that 14 DEGs were enriched in the flavonoid biosynthesis. Out of these 14 DEGs, 8 were downregulated in ZS, whereas 6 were upregulated in ZS (Fig. 5A). Among the upregulated genes in LSZ are *CYP73A16* (trans-cinnamate 4-monooxygenase), *CYP75B2* (flavonoid 3’-hydroxylase), *HST* (shikimate O-hydroxycinnamoyltransferase), *DFR* (dihydroflavonol reductase 1), and *CHS2* (chalcone synthase 3) (Figs. 5A and 6). Meanwhile, genes such as *HST* (fatty alcohol: caffeoyl-CoA acyltransferase), *BAHD1* (vinorine synthase), etc., were downregulated in LSZ leaves (Fig. 5A). In the phenylpropanoid biosynthesis pathway, 25 DEGs were enriched between LSZ and ZS. As shown in Figs. 5B and 9 DEGs were downregulated, whereas 16 DEGs were upregulated in ZS. Genes including *CYP84A1* (cytochrome P450 84A1), *PER10* (peroxidase 10), *ALDH2C4* (aldehyde dehydrogenase family 2 member C4), *COMT1* (caffeic acid 3-O-methyltransferase 1 and caffeic acid 3-O-methyltransferase), *GT5* (anthocyanidin 3-O-glucosyltransferase 5-like, partial), *CYP73A16* and others were downregulated in ZS (Fig. 5B). However, genes such as *PER54* (peroxidase A2), *4CLL7* (4-coumarate-CoA ligase 1), *COMT1* (XP_010112212.1 caffeic acid 3-O-methyltransferase), *MEE23* (berberine bridge enzyme-like 15), *PER21* (peroxidase 21), *PER64* (XP_010113299.1 peroxidase 64), etc., were upregulated in ZS (Fig. 5B). Correlation analysis of some significant flavonoid biosynthesis genes with flavonoid content in ZS and LSZ reveals that total flavonoid content correlated positively with *CYP73A16*, *CYP75B2*, and *DFR* genes in LSZ, but flavonoid content correlated negatively with *CYP73A16* and *DFR* in ZS (Fig. 1D).

### Analysis of differentially expressed transcription factors and RT-qPCR validation of RNA-seq

From the transcriptomic data, 54 DEGs of transcription factors were identified (Table 2). Of the 54 TFs, 13 were upregulated, and 41 were downregulated in SZ. Among these TF families, the most highly expressed are the *MYB* (9 genes), *ERF* (9 genes), *bHLH* (4 genes), and *WRKY* (4 genes) families (Table 2). Validation of the RNA-seq by RT-qPCR showed that the data are reliable, as the RNA-seq results correlate with RT-qPCR, giving a correlation coefficient (R^2^) of 98% (Fig. 2D).

**Table 2:** Differentially expressed transcription factors in the LSZ-vs-ZS comparison

Gene ID	log2(fc)	Symbol	Description
ZZB_Monoploid.003555	3.95	*ORG2*	Transcription factor ORG2
ZZB_Monoploid.022355	3.64	*WRKY51*	Probable WRKY transcription factor 51
ZZB_Monoploid.020515	2.72	*TIF3B1*	Eukaryotic translation initiation factor 3 subunit B
ZZB_Monoploid.010896	2.02	*BBM2*	AP2-like ethylene-responsive transcription factor
ZZB_Monoploid.006982	1.89	*bHLH10*	Transcription factor bHLH91
ZZB_Monoploid.006276	1.50	*MYB1*	Transcription factor MYB1
ZZB_Monoploid.001133	1.50	*MYB82*	Transcription factor MYB82
ZZB_Monoploid.005746	1.22	*At1g61730*	Probable transcription factor At1g11510
ZZB_Monoploid.023615	1.15	*TAF6*	Transcription initiation factor TFIID subunit 6
MSTRG.13,525	1.10		Nuclear transcription factor Y subunit C-3
ZZB_Monoploid.023552	1.05	*HHO3*	Transcription factor HHO3
ZZB_Monoploid.009463	1.01	*HSFB4*	Heat stress transcription factor B-4
ZZB_Monoploid.006212	1.01	*MYB26*	Transcription factor MYB26
ZZB_Monoploid.001628	-1.04	*WRKY11*	Probable WRKY transcription factor 11
ZZB_Monoploid.020592	-1.09	*ERF6*	Ethylene-responsive transcription factor 6
ZZB_Monoploid.017889	-1.20	*ERF053*	Ethylene-responsive transcription factor ERF054
ZZB_Monoploid.022987	-1.24	*WER*	Transcription factor WER
ZZB_Monoploid.018575	-1.27	*MADS3*	MADS-box transcription factor 17 isoform X1
ZZB_Monoploid.015490	-1.30	*WRKY41*	WRKY transcription factor 46-like protein
ZZB_Monoploid.012707	-1.46	*MADS6*	Truncated transcription factor CAULIFLOWER D, partial
ZZB_Monoploid.010427	-1.47	*At3g18960*	B3 domain-containing transcription factor VRN1
ZZB_Monoploid.006274	-1.48	*MYB1*	Transcription factor MYB30
ZZB_Monoploid.018733	-1.49	*ERF2*	Ethylene-responsive transcription factor 2
ZZB_Monoploid.002646	-1.49	*VRN1*	B3 domain-containing transcription factor VRN1-like
ZZB_Monoploid.014495	-1.51	*BEE1*	Transcription factor BEE 1 isoform X1
ZZB_Monoploid.014221	-1.59	*TCP12*	Transcription factor TB1
ZZB_Monoploid.005720	-1.62	*ETC1*	MYB-like transcription factor ETC1
ZZB_Monoploid.017157	-1.62	*MYB4*	Transcription factor MYB14
ZZB_Monoploid.004990	-1.70	*bHLH53*	Transcription factor bHLH52
ZZB_Monoploid.021023	-1.70	*MYB3R3*	Transcription factor MYB3R-3
ZZB_Monoploid.020594	-1.78	*ERF1A*	Ethylene-responsive transcription factor 2
ZZB_Monoploid.008575	-1.88	*CGA1*	Putative GATA transcription factor 22
ZZB_Monoploid.016220	-1.89	*ERF13*	Ethylene-responsive transcription factor 13
ZZB_Monoploid.020825	-1.99	*WRKY53*	Probable WRKY transcription factor 53
ZZB_Monoploid.015714	-2.00	*ERF017*	Ethylene-responsive transcription factor ERF017
ZZB_Monoploid.011546	-2.06	*HSFA6b*	Heat stress transcription factor A-6b
ZZB_Monoploid.021082	-2.26	*bHLH36*	Transcription factor bHLH36
ZZB_Monoploid.011296	-2.35	*RAP2-2*	Ethylene-responsive transcription factor ERF071
ZZB_Monoploid.012521	-2.51	*ERF109*	Ethylene-responsive transcription factor ERF109
ZZB_Monoploid.005894	-2.57	*WIN1*	EXB67303.1 Ethylene-responsive transcription factor WIN1
ZZB_Monoploid.023330	-2.71	*AP1*	Truncated transcription factor CAULIFLOWER A isoform X1
ZZB_Monoploid.020704	-3.00	*MYC2*	Transcription factor MYC2
ZZB_Monoploid.021053	-3.39	*ERF025*	Ethylene-responsive transcription factor ERF027
ZZB_Monoploid.016221	-3.45	*ERF13*	Ethylene-responsive transcription factor 13
ZZB_Monoploid.000858	-3.56	*At1g50680*	AP2/ERF and B3 domain-containing transcription factor At1g50680
ZZB_Monoploid.013748	-4.33	*bHLH162*	Transcription factor bHLH162
ZZB_Monoploid.022599	-4.77	*HEC3*	Transcription factor HEC3
ZZB_Monoploid.023329	-5.35	*MADS2*	Truncated transcription factor CAULIFLOWER D isoform X1
ZZB_Monoploid.023215	-5.63	*MYB106*	Transcription factor MYB106
ZZB_Monoploid.020913	-9.26	*MYB61*	Transcription factor MYB61
ZZB_Monoploid.002614	-10.14	*MYB14*	Transcription factor MYB4
ZZB_Monoploid.014988	-10.60	*MADS3*	MADS-box transcription factor 6
ZZB_Monoploid.012708	-12.85		MADS-box transcription factor 6-like
ZZB_Monoploid.021736	-13.81		B3 domain-containing transcription factor VRN1

Negative and positive values are the log2 of the fold change between LSZ-vs-ZS comparison. The opposite of the expression in ZS is the LSZ, due to a one-level comparison of LSZ vs. ZS

*LSZ; *Lv Shenzi*, ZS; *Zhong Shen 1 Hao

### Virus-induced gene silencing of the *MaCYP73A16* and the effect on flavonoid biosynthesis after silencing

**Fig. 7:**
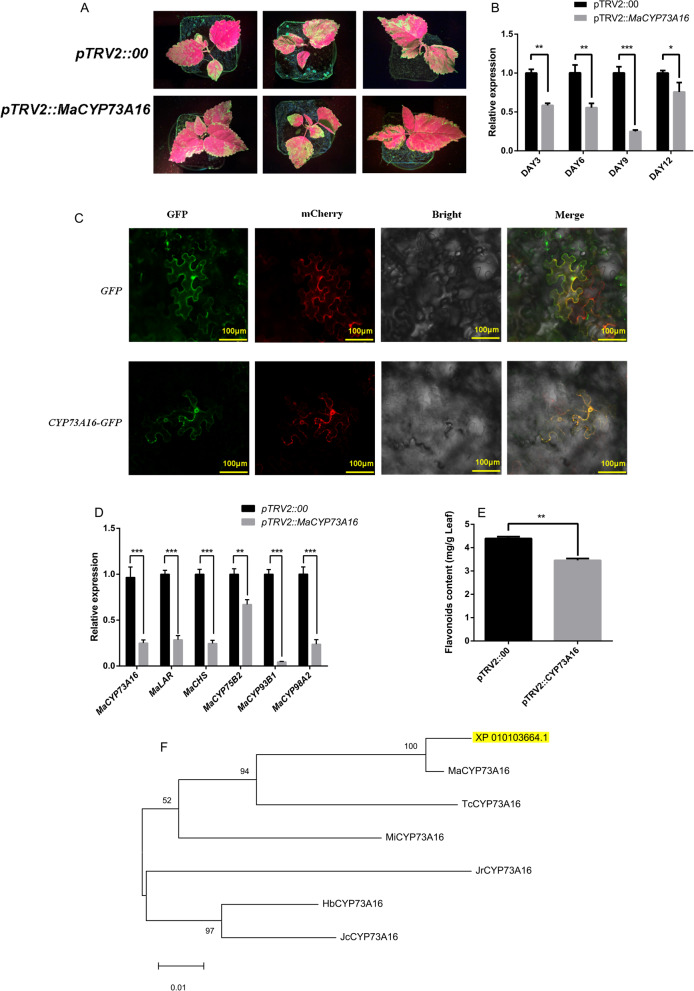
Functional validation of the *CYP73A16* gene. **A** Fluorescence detection results of mulberry leaves (pTRV2-GFP; empty vector control group and pTRV2-GFP- CYP73A16 silencing vector infection group). **B** Relative expression level of the *CYP73A16* gene after silencing. **C** Subcellular localization of MaCYP73A16 in tobacco leaves. Scale bar = 100 μm. **D** Relative expression levels of the *CYP73A16* downstream genes after *CYP73A16* silencing at 9 days. **E** Total flavonoid content after *CYP73A16* gene silencing at 9 days. **F** Phylogenetic relationship of CYP73A16 protein (XP_010103664.1, query sequence in yellow) with other plants (MaCYP73A16 (*M. alba*), MiCYP73A16 (*M. integrifolia*), HbCYP73A16 (*H. brasiliensis*), JrCYP73A16 (*J. regia*), TcCYP73A16 (*T. cacao*), JcCYP73A16 (*J. curcas*). Columns are the mean values of three replicates, and error bars represent the standard deviation of the three replicates. Asterisks represent statistical significance at * *p* < 0.05; ** *p* < 0.01, *** *p* < 0.001 using the Student’s t-test. *CYP93B1* (Licodione synthase), *CYP98A2* (coumaroyl ester 3’-hydroxylase), *LAR* (Leucoanthocyanidin reductase), *CYP75B2* (flavonoid 3’-hydroxylase), *CHS* (chalcone synthase), *CYP73A16* (Trans-cinnamate 4-monooxygenase)

Successful Agrobacterium infection of mulberry seedlings was confirmed by leaf fluorescence (Fig. 7A). Leaf samples were collected at 3, 6, 9, and 12 days post-infection. qRT-PCR analysis of the *MaCYP73A16* gene revealed that expression in the empty vector control group (pTRV2-GFP) remained stable and comparatively high across all time points (Fig. 7B). In contrast, the silencing vector group (pTRV2-GFP-CYP73A16) showed a progressive decline in *MaCYP73A16* expression from days 3 to 9, with maximum silencing efficiency and the lowest relative expression achieved on day 9. Subcellular localization analysis shows that the fluorescence co-expression of the marker gene and the target gene *MaCYP73A16* results in overlaps in the merge channel, indicating that the localization of *MaCYP73A16* matches the prediction and is located in the endoplasmic reticulum (ER) (Fig. 7C).

Further analysis was conducted to assess the impact of silencing *MaCYP73A16* on its downstream genes. As shown in Fig. 7D, the silencing of *CYP73A16* reduced the relative expression of the downstream genes *CYP93B1* (licodione synthase), *CYP98A2* (coumaroyl ester 3’-hydroxylase), *LAR* (leucoanthocyanidin reductase), and *CHS*, except for *CYP75B2* (flavonoid 3’-hydroxylase), whose expression increased. Meanwhile, the most affected gene was *CYP93B1* (Fig. 7D). To further understand the influence of *CYP73A16* silence on flavonoid content in mulberry, leaf samples during the 9-day silence period were used to determine flavonoid content. As illustrated in Fig. 7E, total flavonoid content reduced significantly (*p* ≤ 0.05) in silencing leaves compared to the leaves containing the empty vector, suggesting that the *CYP73A16* gene plays a key role in flavonoid biosynthesis in the mulberry plant.

## Discussion

Flavonoids, a major class of polyphenolic secondary metabolites in mulberry leaves, possess significant biological activities [23, 44]. They influence fruit color and flavor, enhance plant resilience against stresses, and provide key pharmacological functions, including anti-cancer, hypoglycemic, anti-viral, anti-inflammatory, cardiovascular, and anti-Alzheimer’s effects [9, 10]. The flavonoid composition, crucial for silkworm nutrition and human health, is highly genotype-dependent. For instance, varietal differences profoundly affect the content and proportion of specific flavonol glycosides [44]. This genotypic variation underpins the accumulation of diverse flavonoid structures. Driven by demands for healthy silkworm feed and traditional medicine, interest in the nutritional and health benefits of mulberry is growing. However, despite established knowledge of flavonoid benefits and biosynthesis, research gaps remain regarding the differential transcriptomic responses induced by mulberry genotype and variety, which, in turn, prompted the genesis and basis of this study.

The differential abundance of DEGs is significant for metabolite accumulation and the varied influx of flavonoids, as these genes activate diverse biosynthetic pathways influenced by plant cultivar and variety. For instance, a transcriptomic analysis of jujube fruit across developmental stages identified 107, 101, 98, 95, and 96 DEGs at successive phases, linking decreased flavonoid content to the downregulation of specific structural genes [23]. In the present study, while 1124 DEGs were downregulated and 814 were upregulated in cultivar ZS, the opposite pattern was observed in LSZ (Fig. 2A). This supports the hypothesis that variations in flavonoid content arise not only from the regulatory patterns of biosynthetic genes but also from the quantity of structural and regulatory DEGs induced in each cultivar, leading to divergent accumulation and pathway activity. This premise is confirmed by the significant variation in flavonoid accumulation between LSZ and ZS (Fig. 1C), where LSZ exhibited a 38% increase in total flavonoid content relative to ZS. These differential gene expression patterns, which directly correlate with differences in flavonoid content, concurrently activated multiple interconnected pathways, including flavonoid and phenylpropanoid biosynthesis and broader secondary metabolite biosynthesis, while also inducing various pathway-specific transcription factors (Fig. 4A-B). The co-activation of the flavonoid and phenylpropanoid pathways is critical, as the latter serves as the essential upstream precursor pathway feeding into flavonoid synthesis. The two cultivars exhibited distinct expression profiles for key structural genes. Within the flavonoid biosynthesis pathway, genes such as *CYP73A16*, *CYP75B2*, *HST*, *DFR*, and *CHS2* (Figs. 5A and 6) were significantly upregulated in LSZ but downregulated in ZS. This may indicate that the reduced flavonoid content in ZS is a direct consequence of diminished activity in these core biosynthetic genes. A parallel expression pattern was observed for genes within the phenylpropanoid pathway (Fig. 5B). Recent research has characterized two pivotal polyketide synthases in mulberry, *MnCHS* and *MnSTS* [45]. *MnCHS* catalyzes the formation of naringenin and facilitates the novel biosynthesis of steppogenin, while *MnSTS* produces resveratrol and oxyresveratrol, underscoring their fundamental roles in these metabolic routes. Furthermore, master regulatory transcription factors (TFs; a.k.a. regulatory genes) exhibited corresponding expression differences. Transcription factors, including *MYC*-like, *bHLH*, R2R3-*MYB*, and *WDR*, are known to play a key role in flavonoid biosynthesis [20, 21, 46]. For instance, in this study, key TFs, including *MYB4*, *MYB61*, *MYB106*, *bHLH162*, *bHLH36*, and *WRKY41* (Table 2), were expressed at significantly higher levels in LSZ, suggesting that they may promote the activation of downstream flavonoid genes. Consequently, the coordinated downregulation of these TFs and their target structural genes in ZS may have directly resulted in its reduced flavonoid accumulation, robustly supporting the hypothesis that intrinsic genetic variation is the primary determinant of differential flavonoid content in mulberry.

Within the flavonoid biosynthetic network of mulberry, the interplay of structural enzymes signifies the existence of alternative metabolic routes involving distinct genes. For example, the activation of core flavonoid genes such as *PAL*, *CHS*, *CHI*, *F3H*, *F3’H*, *F3’5’H*, and *DFR*, alongside specific *CYP450* gene families, characterizes parallel and alternative pathways. Functionally, *MnCHS* catalyzes the transformation of 2,4-dihydroxycinnamoyl-CoA into the chalcone steppogenin, while *MnSTS* hydrolyzes substrates including p-coumaroyl-CoA and 2,4-dihydroxycinnamoyl-CoA into resveratrol and oxyresveratrol, respectively [45]. This direct one-step synthesis of oxyresveratrol from 2,4-dihydroxycinnamoyl-CoA demonstrates an alternative route that bypasses typical CYP450 enzymes. Conversely, the *CYP75* gene family, encoding *F3′H* and *F3′5′H*, is crucial for hydroxylating the flavonoid B-ring, a key step in cyanidin and delphinidin biosynthesis, the precursors for red and blue anthocyanins [23, 47].

To confirm the role of *CYP450* genes identified via transcriptome analysis in mulberry flavonoid biosynthesis, we characterized the *MaCYP73A16* gene using silencing analysis in mulberry. CYP450 genes are well-documented in flavonoid biosynthesis across species. In mulberry, *MaCYP71BG22* catalyzes the stereoselective hydroxylation of (R)-2-methylpiperidine to synthesize (2R, 4R)-2-methylpiperidin-4-ol [25]. Similarly, *GmCYP93B16* functions as a flavone synthase II in soybean [48]. Strikingly, in mulberry, overexpression of *MaCYP71BG22* increased 1-deoxynojirimycin (DNJ) content while virus-induced gene silencing reduced it, underscoring the functional importance of this family [25]. In this study, silencing *MaCYP73A16* reduced the expression of downstream genes, including *MaCYP98A2*, *MaLAR*, *MaCHS*, and most notably *MaCYP93B1* (Fig. 7B-D). While the VIGS experiments were conducted in *M. alba* car. Yu‑711 rather than the original ZS and LSZ cultivars, the conserved flavonoid pathway across mulberry species, and the consistent expression of *CYP73A16* in Yu-711, and the closeness of CYP73A16 in the ZS and LSZ to Yu-711 via phylogenetic (Fig. 7F) analysis, support the functional relevance of *MaCYP73A16*. This silencing concurrently caused a significant reduction in total flavonoid content, likely by limiting the availability of oxygen and NADPH as catalytic substrates. This aligns with reported functions, in which CYP450 genes catalyze key steps, such as the hydroxylation of (R)-2-methylpiperidine [25]. Furthermore, genes in the *CYP93C* subfamily are involved in the biosynthesis of the isoflavonoid skeleton via hydroxylation and aryl migration [49]. Supporting evidence from *Pyrus spp*. shows that silencing Pbr031195-v2.1 decreased flavonoid content and gene expression, while overexpression increased both [21], confirming the conserved role of CYP450s. Correlation analysis revealed a strong positive relationship between flavonoid content and key flavonoid biosynthesis genes, including *CYP73A16*, *CYP93B1*, and *DFR*, in LSZ, but the reverse surfaced in ZS (Fig. 1D), validating the involvement of *CYP73A16* as a key gene in the biosynthesis of flavonoids. Intriguingly, the downstream gene *MaCYP75B2*, which encodes flavonoid 3’-hydroxylase, shows relatively high expression after *MaCYP73A16* silencing (Fig. 7D). This may result from genetic redundancy and could explain the slight residual flavonoid content observed post-silencing, warranting further investigation. We acknowledge that the VIGS validation ought to have been conducted using ZS and LSZ rather than Yu-711. However, the limited study materials for ZS and LSZ (including no mutants generated yet and no seeds available to produce seedlings for a VIGS experiment) pose a limitation for our current study.

## Conclusion

In summary, this study presents the first transcriptome profiling of the mulberry cultivars Zhong Shen 1 Hao and Lv Shenzi. Integrated physiological and transcriptomic analyses reveal differential flavonoid contents and gene expression, and distinct sets of differentially expressed genes involved in flavonoid and phenylpropanoid biosynthesis between the two cultivars, confirming our hypothesis that inherent genetic variation among diverse mulberry cultivars underlies the differential flavonoid content observed in our study. Silencing *MaCYP73A16* significantly reduced both flavonoid content and downstream gene expression, with peak silencing efficiency observed on the ninth day. These findings provide mechanistic insight into the differential accumulation of flavonoids in mulberry cultivars.

## Supplementary Information

Supplementary Material 1.

## Data Availability

Data used in this work are described in the article, and additional data are provided as supplementary materials. Transcriptome raw data were deposited at the NCBI Sequence Read Archive (SRA) (Accession number: PRJNA1373507) and available online under the link (https://www.ncbi.nlm.nih.gov/sra/PRJNA1373507).
