# Supplementary material for: Transcriptomics and functional characterization identify CYP73A16 as a key regulator of flavonoid biosynthesis in mulberry leaves
Source: BMC Plant Biol. 2026 May 2;26:1152. doi: 10.1186/s12870-026-08804-3 (PMC13348818; doi:10.1186/s12870-026-08804-3)
Supplement: Supplementary file 1 — Supplementary Material 1. [file 12870_2026_8804_MOESM1_ESM.zip › Supplementary materials/Supplementary Figures.docx]

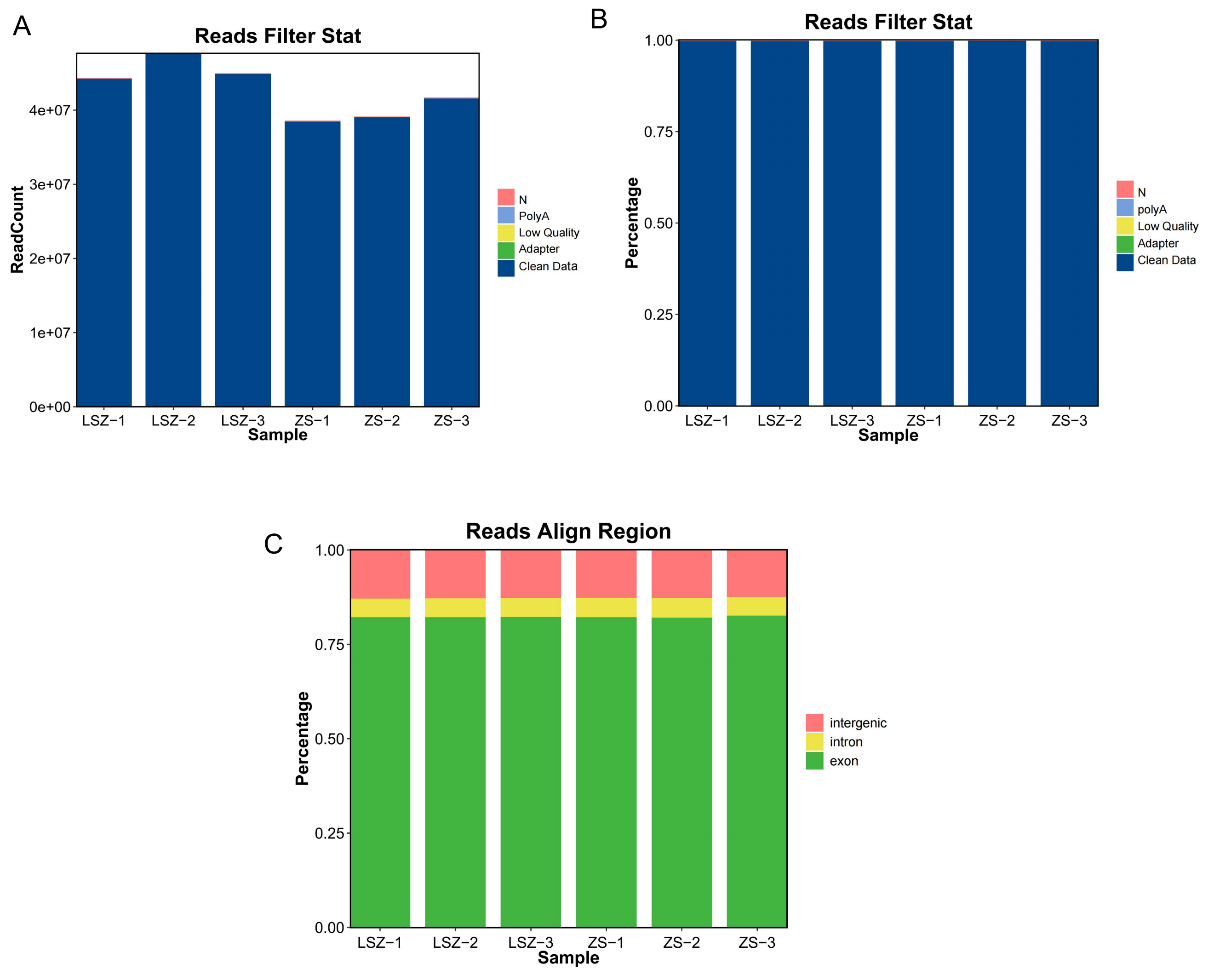


**Fig. S1**. Sequence statistics of the transcriptome data. (A) Reads count filtering. (B) Read filtering (%). (C) Reads regional alignment (%) to reference genome. Clean data (%): the percentage of raw reads remaining after removing low-quality reads, adapters, poly-A sequences, and other contaminants. Low quality (%): the percentage of reads discarded due to poor base quality scores. Adapter (%): the percentage of reads containing adapter sequences (artificial DNA sequences added during library preparation). poly A (%): the percentage of reads consisting primarily of poly-A tails (strings of adenine nucleotides). N (%): the percentage of reads containing ambiguous bases. LSZ: Lv Shenzi. ZS: Zhong Shen 1 Hao.


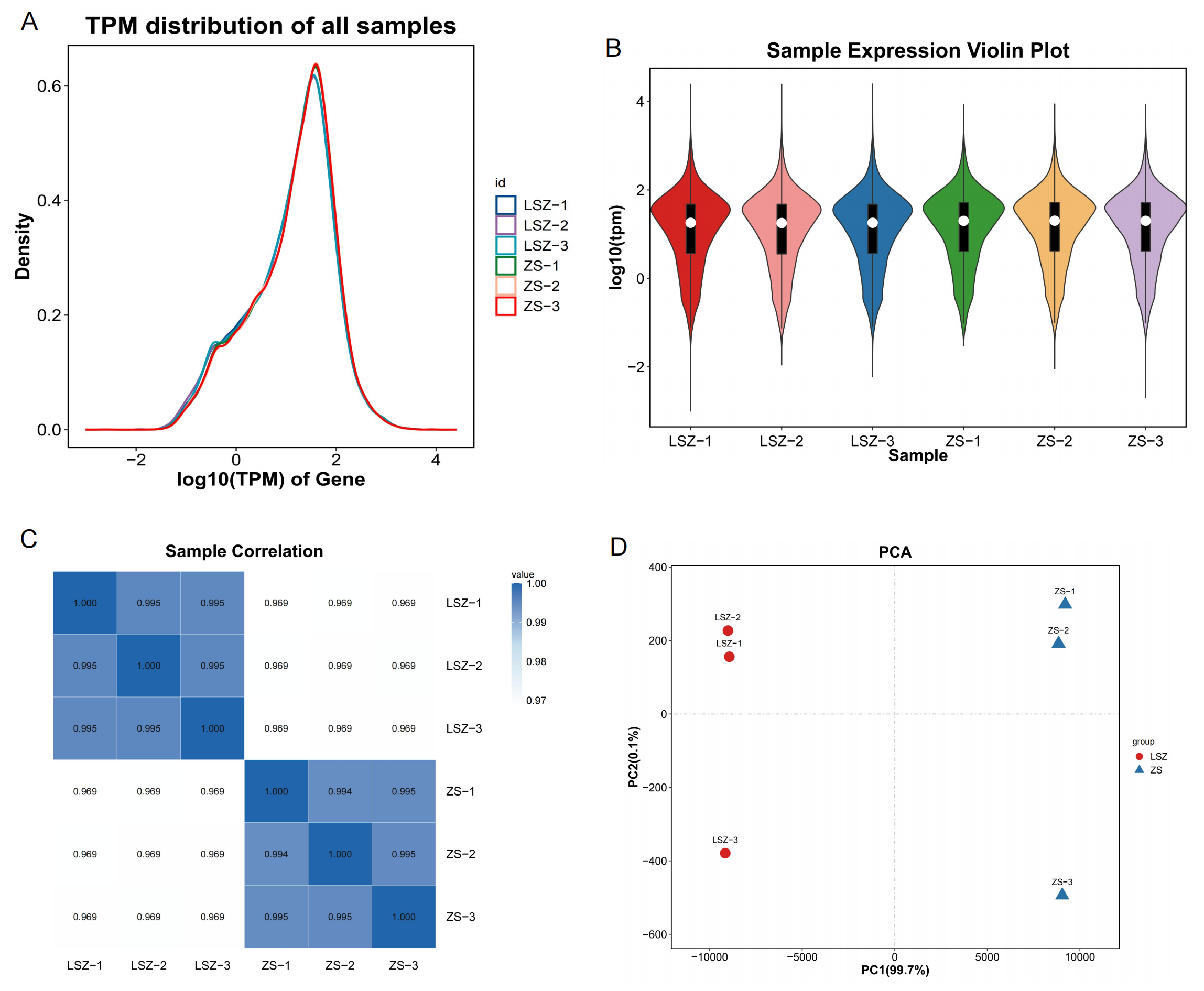


**Fig. S2.** (A) Density plot of TPM (transcript per million mapped reads). (B) Sample gene expression distribution. (C) Sample correlation heatmap analysis. (D) Principal component (PC) analysis. LSZ: Lv Shenzi. ZS: Zhong Shen 1 Hao.


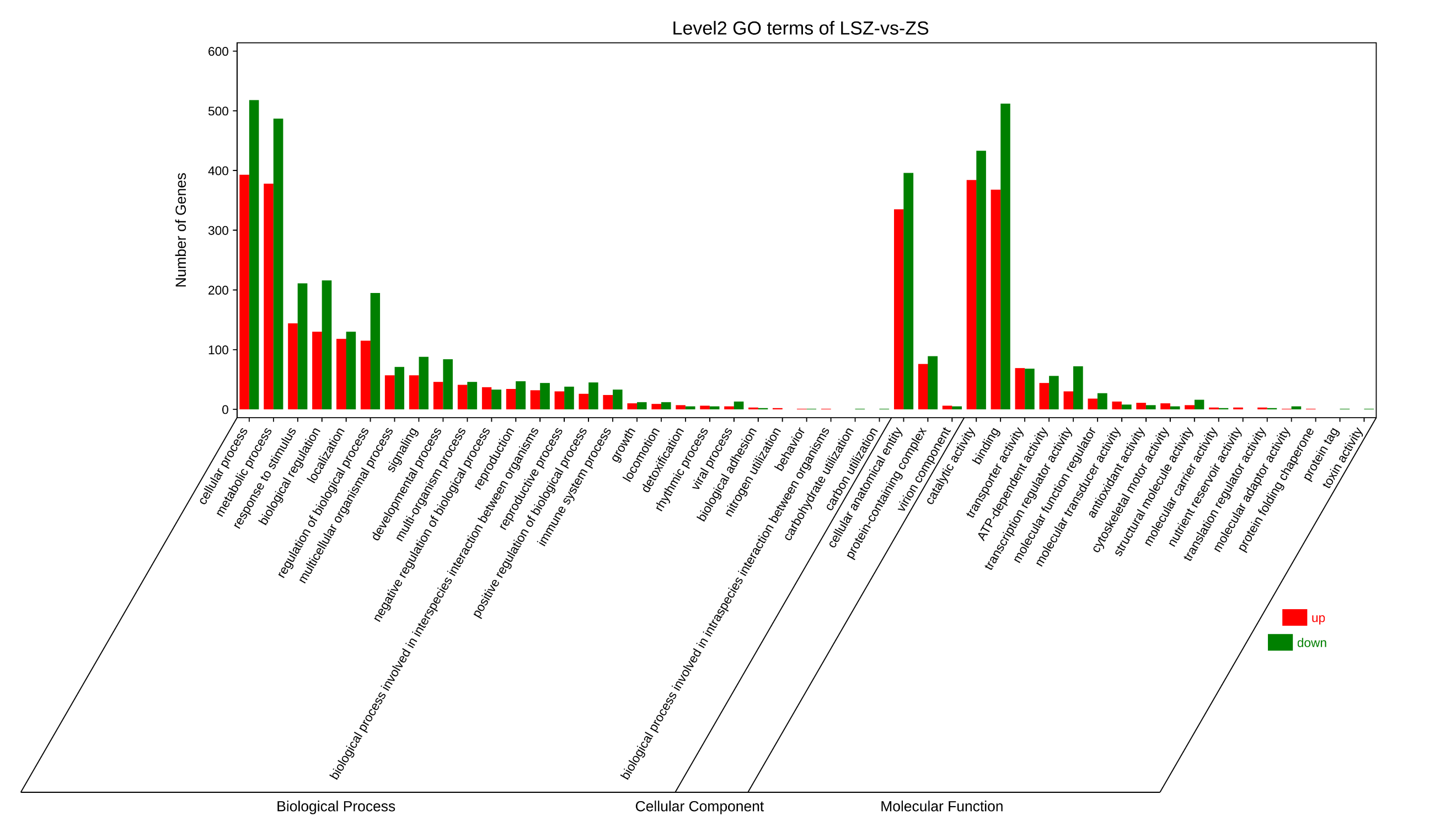


Fig. S3. Gene ontology (GO) secondary classification levels of the up- and downregulated genes.
